# Monocyte Chemoattractant Protein 1 is a Prognostic Marker in ANCA-Associated Small Vessel Vasculitis

**DOI:** 10.1155/2009/584916

**Published:** 2009-07-05

**Authors:** Sophie Ohlsson, Omran Bakoush, Jan Tencer, Ole Torffvit, Mårten Segelmark

**Affiliations:** ^1^Department of Nephrology, Lund University Hospital, Institute for Clinical Sciences, 221 85, Lund, Sweden; ^2^Kidney Research Laboratory, BMC C14, 221 84, Lund, Sweden

## Abstract

*Background*. The (anti neutrophil cytoplasmatic autoantibody ANCA), associated small vessel vasculitides (ASVV) are relapsing-remitting inflammatory disorders, involving various organs, such as the kidneys. (Monocyte chemoattractant protein 1 MCP-1) has been shown to be locally up regulated in glomerulonephritis and recent studies have pointed out MCP-1 as a promising marker of renal inflammation. Here we measure urinary cytokine levels in different phases of disease, exploring the possible prognostic value of MCP-1, together with (interleukin 6 IL-6), (interleukin 8 IL-8) and (immunoglobulin M IgM). *Methods*. MCP-1, IL-6 and IL-8 were measured using commercially available ELISA kits, whereas IgM in the urine was measured by an in-house ELISA. *Results*. The MCP-1 levels in urine were significantly higher in patients in stable phase of the disease, compared with healthy controls. Patients in stable phase, with subsequent adverse events; had significantly higher MCP-1 values than patients who did not. MCP-1 and IgM both tended to be higher in patients relapsing within three months, an observation, however, not reaching statistical significance. Urinary levels of IL-6 correlated with relapse tendency, and IL-8 was associated with disease outcome. *Conclusions*. Patients with ASVV have raised cytokine levels in the urine compared to healthy controls, even during remission. Raised MCP-1 levels are associated with poor prognosis and possibly also with relapse tendency. The association with poor prognosis was stronger for U-MCP-1 than for conventional markers of disease like CRP, BVAS, and ANCA, as well as compared to candidate markers like U-IgM and U-IL-8. We thus consider U-MCP-1 to have promising potential as a prognostic marker in ASVV.

## 1. Introduction

(Antineutrophilic cytoplasmatic autoantibodies, ANCA)-associated small vessel vasculitides (ASVVs) is a group of inflammatory disorders, characterized by inflammation and necrosis of blood vessels and frequent granuloma formation [1]. The patients make autoantibodies against proteins present in the granules of neutrophils and monocytes, mainly proteinase 3 (PR3) and myeloperoxidase (MPO) [2]. The disorders, including Wegener's Granulomatosis (WG) and microscopic polyangiitis (MPA), have a relapsing-remitting progression and a high mortality if untreated. The pathogenesis is still poorly understood. As the ANCA titer correlates with disease activity in some studies [3, 8], a pathogenic role for the autoantibodies has been implicated. Proinflammatory cytokines, such as tumor necrosis factor alpha (TNF-*α*), interleukin (IL)-1*β*, and IL-8, have been found to be elevated systemically and locally at inflammatory sites in WG [9–12]. Moreover, a pathogenic role has been attributed to IL-8 in ANCA-associated glomerulonephritis [10]. In an earlier study, we looked at the circulating cytokine profile in ASVV in stable remission as well as in different degrees of activity [13]. Our main finding was that patients with ASVV have raised circulating cytokine levels, compared to healthy controls, even during stable remission—indicating that there is persistent disease activity, kept under control by inhibitory anti-inflammatory cytokines.

Previous studies have shown that the renal function at diagnosis is a strong predictor not only for renal survival but also for patient survival in ASVV [4, 14, 15]. Other factors have also been reported to predict outcome in ASVV such as severity of the disease at diagnosis, treatment related infections, alpha 1 antitrypsin deficiency, high levels of PR3-ANCA measured by capture ELISA, and low levels of thrombocytes [16–20]. However these findings have usually not been confirmed in repeated investigations. Proteinuria, severe interstitial fibrosis, and glomerulosclerosis which are known to predict outcome in chronic proteinuric glomerulonephritides have also been found to be important risk factors for development of renal failure in ASVV [14, 15, 21, 22]. 

In other glomerulonephritides, not associated with vasculitis, elevated urinary excretion of high molecular weight proteins, for example IgM, has been found to be a better predictor of renal outcome than the degree of albuminuria [23–25]. Tencer et al. reported high urinary IgM excretion in many patients with crescent glomerulonephritis and ASVV [7], and a later study investigated the prognostic significance of urinary IgM excretion in ASVV compared to other known or putative prognostic markers [26]. In conclusion, for patients with ANCA-associated small vessel vasculitis, a high level of urine IgM excretion at time of diagnosis was strongly associated with the development of end stage renal disease, and in addition to old age, also predicted patient survival. 

MCP-1 (CCL2) is a chemokine which specifically attracts blood monocytes and tissue macrophages to its source, via interaction with its cell surface receptor, CCR2 [27]. Renal cells produce MCP-1 in response to various pro inflammatory stimuli, and the expression has been shown to be locally up regulated in glomerulonephritis [28] as well as in diabetic nephropathy [29]. Indirectly, by macrophage recruitment, and also via direct induction of a fibrotic response in glomerular mesangial cells—MCP-1 has the potential to drive the process of renal fibrosis [30]. There are several studies on MCP-1 in diabetes and diabetic nephropathy [30], as well as in SLE [31], whereas there is so far only one clinical study exploring the significance of this protein in ASVV [5]. Other cytokines, like IL-8 and IL-6 have been more thoroughly investigated, and U-IL-8 has been suggested as a candidate marker of renal involvement [10]. 

In this study we measured the levels in urine of MCP-1, IL-8 and IL-6, together with IgM, in well-characterized patients suffering from ASVV, with the aim to explore a possible prognostic value, as compared to more conventional markers of disease.

## 2. Methods

### 2.1. Patients

Between February 2001 and March 2003, all patients with ASVV, according to the Chapel Hill Consensus Conference disease definition, visiting the department of Nephrology, Lund University Hospital, were invited to be included. Patients with dialysis treatment or cancer were not included. No samples were taken from patients when having ongoing viral or bacterial infections. The patients were followed during four to six years by regular visits at the clinic. Clinical status, (Birmingham vasculitis activity score BVAS), relapses as well as the development of any critical damage (damage consistent with significant organ failure) according to the (vasculitis damage index VDI) or death due to vasculitic complications was registered [32]. The patients' status at time of sampling was classified as remission (BVAS 0-1), chronic grumbling activity (BVAS < 5) or relapse/new disease activity (BVAS > 6). These observations were made without access to the results of our analyses. The data presented in tables and figures here in after are based on the index sample of each patient, taken at inclusion in the study. When looking at the possible connection between U-MCP1 levels and prognosis or relapse, only patients in stable phase of disease (remission or chronic grumbling disease) were included. The control group consisted of healthy blood donors (HBDs). These studies were performed with the permission of the Ethical Committee, Lund University, and the written informed consent of the patients. See Table 1 for further patient characteristics.

### 2.2. ELISA Measurements of IL-6, IL-8, and MCP-1 in Plasma and Urine Samples

The blood samples were taken in EDTA tubes and immediately centrifuged, and hence plasma was taken off and stored in −20°C. Portions of 30 mL first voided urine were collected in polyethylene vessels (Kebo AB, Sweden). After the addition of 1 mL of preservation solution (containing benzamidinium chloride, EDTA, tris (hydroxymetyl)–aminoethane and azide; resulting in stable levels of proteins in frozen urine samples, [6]), the urine samples were kept frozen at −20°C until assayed. 

A quantitative sandwich enzyme immunoassay from R&D systems (Abingdon, UK), where a monoclonal antibody specific for IL-6, IL-8, or MCP-1 had been pre-coated onto a microplate, was used. Briefly, Assay Diluent RD1A and Standard or sample was added to each well and left to incubate for two hours at room temperature. The plates were washed four times, to eliminate any unbound substances. Then Conjugate (a polyclonal antibody conjugated to horseradish peroxidase) was added to each well for detection of the cytokine. After two hours' incubation at room temperature, the plates were washed four times and Substrate Solution was added to each well. 20 minutes incubation at room temperature allowed color development in proportion to the amount of cytokine bound in the initial step. Finally, Stop Solution was added to each well and the intensity of the coloring measured. The absorbance was read at 450 nm and 570 nm (correction wavelength).

### 2.3. U-IgM

Urine IgM was measured by an ELISA technique described in detail earlier [6]. A polyclonal sheep anti-IgM antibody was used as capture antibody, and an AP-conjugated goat antihuman IgM antibody was used for detection.

### 2.4. Cystatin C, CRP, Creatinine, Protein HC

P-Cystatin C was measured as marker of glomerular filtration and was used for correction of cytokine levels in plasma [13]. C-reactive protein and creatinine are conventional markers of inflammation and renal function, respectively. Creatinine was measured in both serum and urine. Protein HC was also detected in urine. 

The Clinical Chemical Laboratory at Lund University Hospital, Lund, Sweden, performed analyses on a Hitachi 917 Pluto. Kits from Roche Diagnostics and Dako were used. 

Plasma cystatin C and urine protein HC were determined by immunoturbidimetry, and plasma and urine concentrations of creatinine were measured by use of a creatininase enzyme-based procedure.

### 2.5. ANCA

Wieslab AB, Lund, Sweden, performed analyses of PR3-ANCA and MPO-ANCA by routine methods [33, 34].

### 2.6. Statistical Analysis

All statistics were performed in StatView 5.01. For correlation analysis, the nonparametric Spearman's rank correlation test was used in order to reduce the impact of outliers. Simple regression analysis was performed on logarithmic values, for normally distributed parameters. Analysis of variance was done using the nonparametric Kruskal-Wallis test and Man Whitney U-test.

## 3. Results

In all 82 patients with stable ASVV were included in the study. At inclusion 57 were in remission, while 25 had grumbling disease activity. Samples at time of relapse were collected from an additional 17 patients—these were not included when looking at the potential relation between U-MCP-1 levels and outcome/relapses (Table 1). Severe outcome, as defined in the methods section, occurred in 15 patients. During followup 23 patients developed at least one relapse, in 12 cases samples were taken within three months before the clinical relapse. Overall there were no statistical differences whatsoever between patients with different ANCA specificity. Subgrouping according to ANCA specificity was thus considered superfluous, and these data are not shown.

### 3.1. U-MCP-1

**Figure 1:**
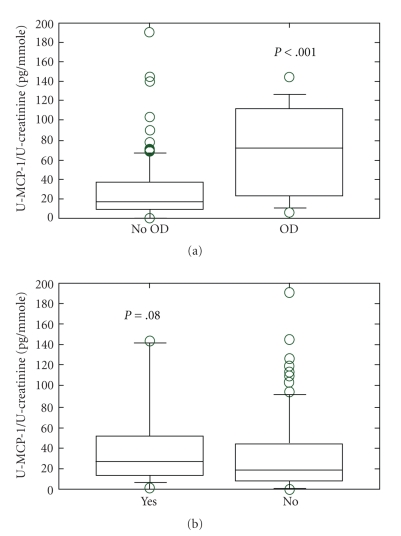
(a) U-MCP-1 as prognostic marker No OD: no development of critical damage according to VDI during followup. OD: development of critical damage according to VDI. All patients were in stable phase of the disease when the sample was taken (7 in remission, 8 with smouldering disease in the group who developed OD; 50 in remission and 17 with smouldering activity in the group who did not develop OD), presented as pg MCP-1/mmole creatinine. The box plot shows median value, with 95% confidence interval. (b) U-MCP-1 as predictor of relapse Yes: relapse within 3 months. No: no relapse within 3 months. All patients in stable phase of the disease when the sample was taken (6 in remission and 6 with smouldering disease in the group with subsequent relapse; 51 in remission and 19 with smouldering activity that did not relapse), presented as pg MCP-1/mmole creatinine. The box plot shows median value, with 95% confidence interval.

The urinary MCP-1 levels were significantly higher in patients in stable phase of the disease (*n* = 82), compared with healthy controls (*n* = 14) (17, undetected-191 versus 10, undetected-29 pg/mmol creatinine, *P* < .05); see Table 2. Patients in stable phase, who developed critical damage or died during the followup had significantly higher MCP-1 values than patients who did not (72, 6.0–145 versus 15, undet-191 pg/mmol creatinine, *P* < .001; *n* = 15, 7 patients in remission and 8 patients with smouldering disease). There was also a tendency toward higher levels in patients relapsing within three months (*n* = 12, 6 patients in remission and 6 patients with smouldering disease), an observation, however, not reaching statistical significance, see Figure 1. Raised U-MCP1 was stronger associated with severe outcome than all of the other markers measured in urine, see Table 3. When dividing the patients in stable phase into two groups with high (defined as > 2 standard deviations above median value) and low U-MCP-1 levels respectively, the positive predictive value for critical damage was 70%. The negative predictive value, that is, no critical damage if the U-MCP-1 level was low, was 89%. 

No correlation could be seen with plasma levels of MCP-1, and there was no significant correlation with CRP, ANCA, BVAS, U-IL6 or U-IgM. A weak positive correlation was seen with U-IL-8 (*r* = 0.3, *P* < .05) and there was a strong positive correlation with U-protein HC (*r* = 0.6, *p* < .0001), indicating a tubular origin, which is consistent with earlier studies [30]. The correlation with the renal function markers in plasma—creatinine and cystatin C—was *r* = 0.2, *P* < .05, and *r* = 0.4, *P* < .01, respectively.

Until now we have repeated measurements on 10 patients, and intra individual variation in U-MCP-1 so far seems small, although a small increase before and during relapse seems to occur. These data are preliminary and not shown. 

Plasma measurements of MCP-1 showed raised levels in patients compared to healthy controls, however this was not significant after correction for renal function (data not shown).

### 3.2. U-IgM

**Figure 2:**
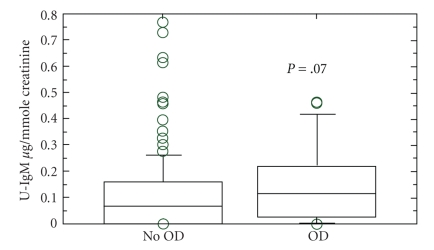
U-IgM as prognostic marker No OD: no development of critical damage according to VDI during followup. OD: development of critical damage according to VDI. All patients in stable phase of the disease when the sample was taken (7 in remission and 8 with smouldering disease; 50 in remission and 17 with smouldering activity in the group who did not develop OD, presented as *μ*g IgM/mmole creatinine. The box plot shows median value, with 95% confidence interval.

Independent of disease activity, IgM levels in the urine were significantly increased in ASVV, compared to healthy controls (9.0, 5.0–70, versus 70, 1.0–800 *μ*g/mmol creatinine, *P* < .001) see Table 2. U-IgM tended to be higher in patients who subsequently died or developed critical organ damage; see Figure 2. In the subgroup with grumbling disease activity this association was statistically significant. IgM also tended to be higher in patients relapsing within three months, an observation, however, not reaching statistical significance.

### 3.3. U-IL-6 and U-IL-8

Urinary levels of IL-6 and IL-8 were higher than in healthy controls; see Table 2. U-IL-8 tended to be associated with severe outcome, and U-IL-6 was increased in patients with subsequent relapses; see Table 3.

## 4. Discussion

This is the first study to report the prognostic significance of urinary MCP-1 excretion in ASVV as compared to other markers of disease-conventional (CRP, ANCA, creatinine) as well as new candidates (IgM, IL-6, IL-8). ). In our study U-MCP-1 correlates with disease activity, and also seems to be a helpful predictor of poor prognosis. This confirms and extends the findings of Tam et al. [8]. They examined whether U-MCP-1 levels could be used in monitoring patients' response to therapy and concluded that reduction of U-MCP-1 levels was a more useful early laboratory marker of response to therapy than reduction of proteinuria, serum creatinine or ANCA titer [8]. 

There are two main possibilities why raised U-MCP-1 may be associated with adverse outcome. First U-MCP-1 may signal an ongoing sub clinical inflammation that in the long run is detrimental for the patient. An alternate explanation is that U-MCP-1 is a marker of renal tubulointerstitial damage, which correlates to severity of renal disease at onset, which in turn affects long-term prognosis. The correlation with U-PHC and creatinine favors the second explanation, while the correlation with disease activity and favors the first. Furthermore in experimental glomerulonephritis, there is increased glomerular synthesis of MCP-1, and systemic administration of an anti-MCP-1 antibody has been demonstrated to reduce the severity of acute glomerulonephritis and subsequent scarring [35, 36]. In this study MCP-1 in plasma was raised in general in our patients, but when adjusting for renal function this was not significant. There are different possible explanations for this phenomenon. One is that declined renal function is associated with greater loss of MCP-1 in the urine. Another explanation is that correcting for declined renal function is bias, because raised MCP-1 levels are per se contributing to renal damage—meaning that it's hard to discern what the egg is, and what the hen is. MCP-1 in the urine most likely comes from both local production and increased filtration due to raised systemic levels. Increased glomerular synthesis could explain the lack of clear-cut correlation between plasma levels and urinary levels. 

In our previous study of IgM excretion in the urine in patients with ASVV, at onset of disease, there was a strong association with development of end stage renal disease [26]. In this study, we did not specifically look at development of end stage renal disease—but instead looked at the overall development of critical damage due to vasculitis. In this setting, IgM excretion in stable patients was not so strongly associated with severe outcome. This can be explained by the fact that IgM in the urine represents the degree of mechanical glomerular damage, whereas MCP-1 is actively secreted in response to several factors of systemic as well as local origin and has the potential of both indirect and direct toxic influence on the renal tissue. IgM may thus be a better marker of glomerular damage, whereas MCP-1 adds information about ongoing inflammation in the kidney, not only systemically. In stable AASV patients it is also rare with microhematuria, another marker of ongoing glomerular inflammation. The prognostic value of MCP-1 most likely comes from this protein's role in driving the process of fibrosis and scarring—so far only shown in the kidney [30], but logically in other organs too. 

U-IL-8 has been suggested to be of value in assessing disease activity [10], but the results of this study were not convincing. The results for U-MCP-1 and U-IL-8 are consistent with previous studies on other diseases, like lupus nephritis and diabetic nephropathy—indicating the reflection of basic pathophysiological mechanisms, rather than anything else [30, 37]. 

In conclusion urinary excretion of MCP-1 is increased in patients with ASVV. The degree of excretion correlates significantly with patient outcome, considering critical damage or death. The association with poor prognosis was stronger for U-MCP-1 than for conventional markers of disease like CRP, BVAS, and ANCA, as well as compared to candidate markers like U-IgM and U-IL-8. We thus consider U-MCP-1 to be a promising prognostic marker in ASVV, and perhaps this protein is also a future therapeutic target. 

Further studies are needed to evaluate and confirm these data long term and longitudinally, preferably in a bigger patient cohort.

## 5. Summary

Urinary excretion of MCP-1 is increased in patients with ASVV. The degree of excretion correlates significantly with patient outcome, considering critical damage or death. The association with poor prognosis was stronger for U-MCP-1 than for conventional markers of disease like CRP, BVAS, and ANCA, as well as compared to candidate markers like U-IgM and U-IL-8. We thus consider U-MCP-1 to be a promising prognostic marker in ASVV.

## 6. Conflict of Interest

We have had no involvements that might raise the question of bias in the work reported or in the conclusions, implications, or opinions stated.

## Figures and Tables

**Table 1:** Patient characteristics.

	Remission	Smouldering	Relapse
No. of patients	57	25	17
% female	74	86	62
Age	63 (19–88)	65 (26–82)	60 (26–83)
% MPO/PR3/seroneg	28/63/9	35/47/8	24/62/14
% relapse within 3 M	10	24	—
% develop critical OD	12	32	—
BVAS	0 (0-1)	3 (2–5)	6 (2–11)
P-Cystatin C (mg/l)	1.3 (0.83–4.4)	1.12 (0.84–3.06)	1.22 (0.92–2.2)
P-Creatinine (*μ* mol/l)	93 (45–498)	81 (52–317)	90 (61–189)
P-CRP (mg/l)	5 (0–57)	9 (0–61)	14 (5–160)
U-PHC (mg/l)	5.6 (0–73.7)	5.7 (0–37.6)	15.9 (5.1–36.1)

MPO = myeloperoxidase-ANCA positive, PR3 = proteinase 3-ANCA positive, seroneg = seronegative, M = months, OD = organ damage, BVAS = Birmingham vasculitis activity score, CRP = C-reactive protein, PHC = protein HC/*β*2-microglobulin. Age, BVAS, plasma and urine measurements are expressed as “median value (range)”.

**Table 2:** Urine levels of MCP-1, IgM, IL-6 and IL-8. MCP-1 = monocyte chemoattractant protein 1, IgM = immunoglobulin M, IL-6 = interleukin 6, IL-8 = interleukin 8. All data are expressed in relation to U-creatinine. Controls = healthy blood donors.

	Remission	Smouldering	Relapse	Controls
U-MCP-1(pg/mmole)	18.8	13.8	36.0	9.9
	(undetected-191.1)	(undetected-144.3)	(3.8–90.3)	(5.4–29.0)
U-IgM(*μ*g/mmole)	80.0	65.0	60.0	9.0
	(undetected-700.0)	(1.0–800.0)	(1.0–300.0)	(5.0–80.0)
U-IL-6(ng/mmole)	0.9	1.0	Ud	0.7
	(undetected-22.3)	(undetected-5.4)	(undetected-1.6)	(0.4–1.4)
U-IL-8(ng/mmole)	4.0	3.5	4.4	2.4
	(undetected-181.3)	(undetected-216.2)	(undetected-25.7)	(0.4–50.7)

**Table 3:** Statistical plausibility of raised potential markers to be associated with outcome and relapse respectively. Severe outcome defined as critical damage, according to (vasculitis damage index VDI) and death. U = urine, MCP-1 = monocyte chemoattractant protein 1, IgM = immunoglobulin M, IL-6 = interleukin 6, IL-8 = interleukin 8, P = plasma, ANCA = antineutrophil cytoplasmatic autoantibodies, CRP = C-reactive protein, BVAS = Birmingham vasculitis activity score. For statistics, the Man Whitney U-test and Kruskall-Wallis' test were employed.

	Severe outcome	Relapse within 3 months
U-MCP-1	**P** < **.001**	*P* = .08
U-IgM	*P* = .07	*P* = .39
U-IL-6	*P* = .10	**P** = **.04**
U-IL-8	**P** = **.05**	*P* = .2
P-IL-6	*P* = .45	**P** < **.001**
P-IL-8	*P* = .09	*P* = .2
ANCA	*P* = .48	*P* = .09
CRP	*P* = .23	*P* = .68
Creatinine	**P** < **.001**	*P* = .32
Cystatin C	**P** < **.001**	*P* = 0.6
BVAS	*P* = .63	*P* = 0.18

**Table 4:** Correlation matrix. U = urine, MCP-1 = monocyte chemoattractant protein 1, IgM = immunoglobulin M, IL-6 = interleukin 6, IL-8 = interleukin 8, ANCA = anti neutrophil cytoplasmatic autoantibodies, CRP = C-reactive protein, BVAS = Birmingham vasculitis activity score. Markers measured in the urine are expressed in relation to U-creatinine (mmol/l). For statistics, the Spearman's rank correlation test and simple regression analysis were performed.

	BVAS	ANCA	Cystatin C	CRP	U-MCP-1)	U-IgM	U-IL-8
U-IL-6	0.02 ns	0.2 ns	0.2 **P** < **.05**	0.07 ns	0.2 ns	0.05 ns	0.2 **P** < **.05**
U-IL-8	0.05 ns	0 ns	0.2 ns	0.1 ns	**0.3 ** **P** < **.05**	0.1 ns	
U-IgM	0.07 ns	0.06 ns	0.3 **P** < **.001**	−0.03 ns	0.1 ns		
U-MCP-1	0.2 ns	0.06 ns	0.4 **P** < **.01**	0.2 ns			
